# Hospital-related healthcare expenditure of impending versus completed pathological femur fractures: a propensity score matched study of 265 patients

**DOI:** 10.2340/17453674.2025.43479

**Published:** 2025-05-27

**Authors:** Tom M DE GROOT, Michelle R SHIMIZU, David SHIN, Olivier Q GROOT, Stein J JANSSEN, Kevin A RASKIN, Eric T NEWMAN, Marco L FERRONE, Santiago A LOZANO-CALDERON, Joseph H SCHWAB, Paul C JUTTE

**Affiliations:** 1 Department of Orthopedics, Massachusetts General Hospital, Boston, MA, USA; 2 Department of Orthopedics, University Medical Center Groningen, Groningen, The Netherlands; 3 Department of Orthopaedic Surgery, Amsterdam University Medical Center, University of Amsterdam, Amsterdam, the Netherlands; 4 Department of Orthopedic Surgery, Brigham and Women’s Hospital, Boston, MA, USA; 5 Department of Orthopedic Surgery, Cedars-Sinai Medical Center, Los Angeles, CA, USA b; a Shared senior authorship.; b Investigation performed at Massachusetts General Hospital, Boston, United States of America

## Abstract

**Background and purpose:**

The prevalence of metastatic bone disease as well as the accompanying societal costs are expected to increase due to advances in cancer treatment. While the literature suggests that there is economic value in prophylactic stabilization compared with the fixation of completed pathological fractures in long bone metastases, studies are limited by their small sample sizes and insufficient correction for potential confounders. We aimed to evaluate whether prophylactic treatment of an impending femur fracture was associated with lower healthcare costs compared with completed pathologic fractures. We further aimed to compare prophylactic surgical treatment with completed pathological fractures in terms of postoperative complications, discharge disposition, and postoperative length of stay.

**Methods:**

This is a retrospective cohort study with propensity score matching (PSM). We included clinical and financial data for 265 patients who received surgery for impending (n = 161) or completed (n = 104) femoral fractures of metastatic lesions, from 2 affiliated urban tertiary care centers between 2016 and 2020 in the United States. After PSM on 13 variables, including demographics and clinical characteristics, 100 impending fractures were matched with 100 completed fractures. The primary outcome was healthcare costs per episode of care, defined as the total cost from admission to 30 days after discharge.

**Results:**

We found no difference in total cost of care between patients undergoing prophylactic surgical treatment and patients who underwent surgical treatment for a completed pathological fracture (median difference 44 cost-units [CU], 95% confidence interval [CI] –294 to 262). No differences were seen when dividing total cost into cost during hospital admission (median difference –25 CUs, CI –152 to 159) and 30 days following discharge (median difference 31 CUs, CI –74 to 88). Patients with completed pathologic fractures were more often discharged to rehabilitation facilities (57/100, vs 30/100, P < 0.01).

**Conclusion:**

In contrast to earlier findings, we showed no difference in treatment costs between surgical management of impending and completed pathological fractures of femur metastases after adjusting for confounding factors. However, patients with completed pathological fractures were significantly more likely to require discharge to rehabilitation facilities, highlighting potential out-of-hospital costs related to extended rehabilitation, reduced mobility, and loss of independence.

The incidence of bone metastases is expected to rise with increasing life expectancy, early detection, and improved cancer treatments [1]. Pathological fractures are associated with high morbidity and mortality rates [2,3]. When there is an increased risk of fracture, surgical stabilization could be a suitable prophylactic treatment [4]. Previous literature has demonstrated multiple beneficial clinical outcomes of prophylactic treatment over treatment of completed pathological fracture, including improved survival, lower risk of complications, less blood loss, and shorter hospital stays [3,5].

Metastatic bone disease is a notable driver of the cost of cancer treatment, with an estimated cost burden of US$12.6 billion in the United States and is predicted to increase [6]. Therefore, in addition to clinical outcomes, there is value in a more comprehensive understanding of the cost-effectiveness of both treatment options, particularly as healthcare systems aim to allocate resources efficiently and improve policy decisions.

Due to the rising trend in healthcare costs, cost-effective clinical practices with the greatest possible patient outcomes are an increasingly important goal of (cancer) treatment. As rates of metastatic bone disease continue to increase, physicians and hospitals must be aware of the relative costs of different treatment options.

The validity of earlier studies is limited by its small sample size, and neither study fully accounted for possible confounding variables [7].

Therefore, we aimed to compare prophylactic surgical treatment with completed pathological fractures in terms of total cost of care in patients’ femoral metastases. Additionally, we aimed to compare prophylactic surgical treatment with completed pathological fractures in terms of postoperative complications, discharge disposition, and postoperative length of stay.

## Methods

### Study design and setting

The guidelines for institutional review board approval were followed for this retrospective cohort study with PSM. This study was performed by manually reviewing electronic records from 2 affiliated tertiary referral hospitals in the United States of America where the index surgery was performed between January 1, 2016, and December 31, 2020.

The study is reported according to the STROBE guidelines.

### Participants

**Figure 1:**
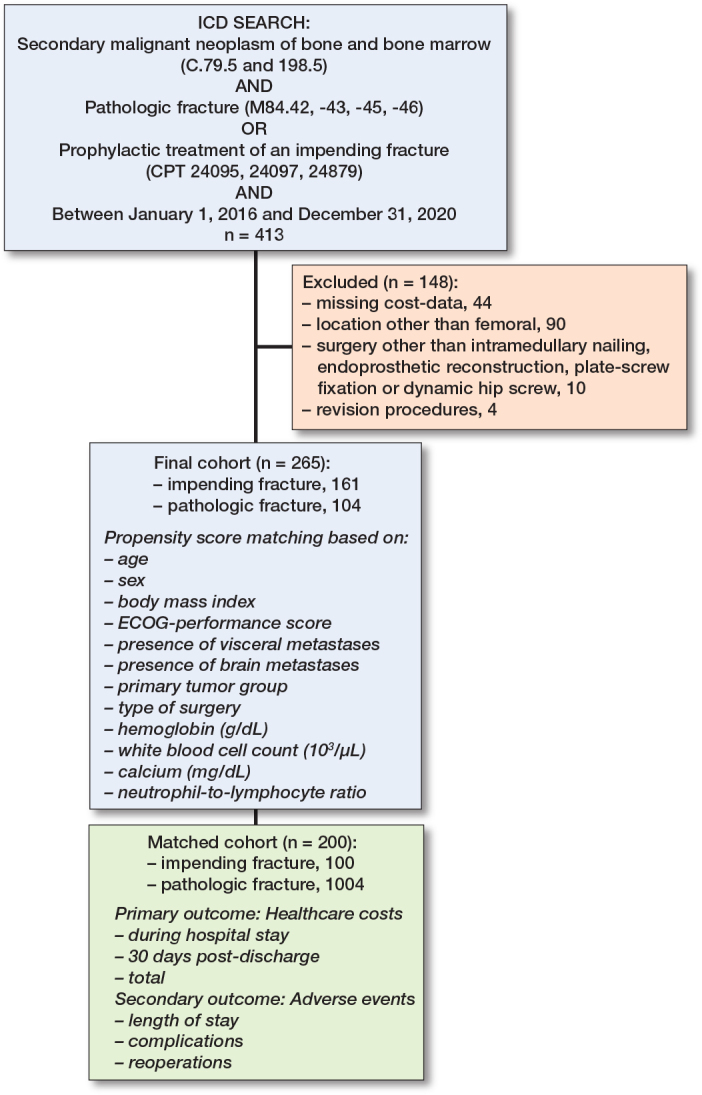
Flowchart of included patients. ICD = International Classification of Diseases; BMI = body mass index; ECOG = Eastern Cooperative Oncology Group .

We included all patients 18 years and older who underwent operative treatment for an impending or completed pathological fracture of the femur. Patients with malignant lymphoma or multiple myeloma were also included due to their comparable treatment approach [8,9]. Exclusion criteria included patients who (i) did not have available cost data, (ii) received surgery other than intramedullary nailing, endoprosthetic reconstruction, or plate–screw fixation, (iii) had incomplete cost data, and (iv) revision procedures (only the initial procedure was considered in patients who underwent multiple surgeries for metastases). Our final cohort consisted of 265 patients who were separated into an impending fracture group (n = 161) and acute completed fracture group (n = 104). After PSM, a balanced cohort consisting of 200 patients was established, including 100 patients in both groups (Figure 1).

### Interventions

Prophylactic surgery was performed in patients who presented with a Mirels score of 8 or higher, indicating a high fracture risk [4]. Similar techniques (intramedullary nailing, endoprosthetic reconstruction, or plate–screw fixation) were used for both impending and completed pathological fractures with the objective of repairing and stabilizing the (impending) fracture. Surgeries were conducted in a tertiary hospital setting by multidisciplinary orthopedic oncology teams consisting of orthopedic surgeons, radiotherapists, medical oncologists, and radiologists experienced in treating patients with metastatic bone disease. Both groups received standardized perioperative surgical care in the affiliated hospitals by the same surgical teams specialized in oncologic orthopedic care.

### Outcomes

The primary outcome was the healthcare costs per episode of care obtained through the institutional financial data for 3 endpoints: (i) during hospital stay, (ii) from the time of discharge to 30 days post-discharge, and (iii) total cost combining the former 2 endpoints. During hospital stay was defined as from the time of admission till discharge. Total cost was defined as the sum of cost during hospital stay and from discharge to 30 days post-discharge. The costs extracted were divided by a common denominator to create a representative cost-unit (CU) value for statistical analysis. A confidence interval excluding differences greater than 10% of cost-units between groups was interpreted as indicating the absence of a clinically meaningful difference [10].

Our secondary outcomes were postoperative complications (recorded within 30 days after discharge: wound infection requiring antibiotics or surgery, wound dehiscence, venous thromboembolism including pulmonary embolism and deep vein thrombosis, urinary tract infections, pneumonia, myocardial infarction, sepsis, delirium, material failure secondary to surgery, and reoperation of index surgery [8,9], postoperative length of stay, and discharge disposition). Most recent follow-up or date of death was used as date of last contact with the patient. The median follow-up time in the non-matched cohort was 298 days (interquartile range [IQR] 120–603). The median follow-up time in the matched cohort was 261 days (IQR 97–561). The last date of follow-up was June 30, 2022. In the first 30 days after discharge, 7/265 (3%) patients were lost to follow-up, all of whom were non-Massachusetts residents.

### Variables

Electronic medical records were manually reviewed to obtain the following baseline variables frequently used in prediction models for extremity metastasis survival prediction [11,12]: age, sex, body mass index (BMI), patient’s general condition assessed through the Eastern Cooperative Oncology Group (ECOG) performance scale [13], any additional Charlson Comorbidity [14], primary tumor location, primary tumor growth type (slow, moderate, or rapid growth) per Katagiri et al. [15], absence or presence of visceral, brain, and/or other bone metastases, type of surgery (intramedullary nailing, endoprosthetic reconstruction, plate–screw fixation, and dynamic hip screw), any previous systematic therapy, and 12 preoperative laboratory values. Patients were indicated to have a comorbidity if they had at least 1 comorbidity listed in the Charlson Comorbidity Index in addition to their metastatic cancer. ECOG performance of each patient was categorized to a scale of either 0–2 or 3–4, where a score of 0–2 represents patients with better performance status, meaning they are more physically able and have fewer restrictions in daily activities. A score of 3–4 represents patients with poorer performance status, who are more limited in their daily activities and more dependent on assistance. Primary tumors were characterized based on their growth type to account for differences in prognosis between different subtypes of tumor origin. For example, this type of grouping allows for the differentiation between patients with breast cancer sensitive or resistant to hormonal therapy. Systemic therapy was defined as having received at least 1 of the following: chemotherapy, immunotherapy, targeted therapy, or hormone therapy. 12 preoperative laboratory values from up to 14 days before the surgery were recorded where the closest to surgery was considered if multiple values were present.

### Sample size

The sample size in this study was inherently limited by its retrospective design, allowing only a finite number of eligible cases to be included. A post-hoc power analysis indicated that, with our sample size of 200, the study achieved a power of 0.80 to detect a statistically significant difference at a 2-tailed alpha level of 0.05 for an effect size of 0.275 using a Mann–Whitney U test. This effect size threshold suggests that the study was adequately powered to detect small-to-moderate differences in the ranks between the groups. The effect size of 0.275 was determined based on Cliff’s Delta, a measure appropriate for non-parametric data [16].

### Propensity score matching

**Figure 2:**
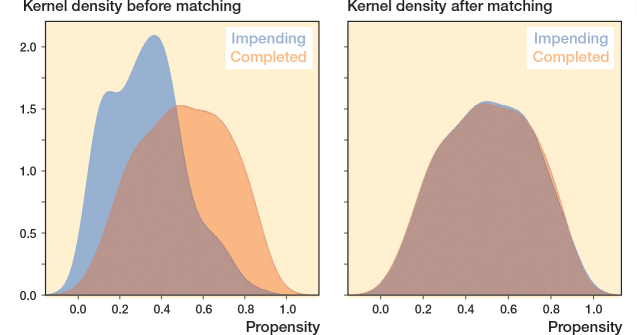
Kernel density plot of propensity score before and after matching.

Propensity score matching (PSM) was used to minimize confounding between surgical treatment of impending vs completed pathological fractures. Based on expert knowledge and the literature, 13 confounding variables were selected to match the 2 groups and ensure comparability (see Figure 1) [17-19]. A one-to-one nearest-neighbor matching was performed in random order, without replacement, using a fixed caliper of 0.046. Calipers up to 0.2 standard deviations of the propensity scores are generally sufficient to retain an adequate sample size for studies with smaller sample sizes [18]. After PSM, a balanced cohort of 200 patients was created including 100 patients in both impending and completed pathologic fracture cohorts. The adequacy of matching was tested by (i) testing the differences of standardized means; (ii) comparing the matched variables with use of standardized mean differences, and (iii) visualization using a kernel-density plot (Figure 2, see Appendix) [19].

### Statistics

Before matching, baseline characteristics between the 2 cohorts were compared using t-test or Mann–Whitney U test for continuous variables and a Fisher’s exact test or a chi-square contingency test for categorical variables. The same tests were also used to compare the non-matched cohort of 265 patients with the 44 excluded patients due to missing cost data to ensure that the excluded patients did not differ significantly in baseline characteristics that could bias the analysis or affect the generalizability of the results. Median costs were assessed using the Mann–Whitney U test and mean costs using the independent Student t-test. Additionally, we calculated median differences (MD) with 95% confidence intervals (CI) to assess the clinical relevance of cost differences between groups. A median difference with a CI excluding zero was considered statistically significant. A confidence interval excluding differences greater than 10% of cost-units between groups was interpreted as indicating the absence of a clinically meaningful difference [10]. The costs extracted were divided by a common denominator to create a representative cost-unit (CU) value for statistical testing.

After PSM, a paired data analysis was performed to evaluate baseline data and outcome differences using the McNemar test for categorical data and the Wilcoxon signed-rank test for continuous data [20].

Missing data were imputed using Missforest imputation [21]. Missing data included BMI: 1 (0.4%); white blood cell count: 33 (12%); hemoglobin level: 33 (12%); platelet level: 34 (13%); creatinine level: 37 (14%); sodium level: 37 (14%); calcium level: 37 (14%); absolute lymphocyte count: 79 (30%); platelet-to-lymphocyte ratio: 78 (30%); neutrophil-to-lymphocyte ratio: 77 (30%); absolute neutrophil count: 79 (30%). Excluded from the analyses were alkaline phosphatase and albumin as they were missing in more than 30% of the values.

Two-tailed P values of < 0.05 were considered statistically significant. Python (version 3.11.6; Python Software Foundation, Wilmington, DE, USA) was used for data analysis, incorporating the pandas (version 2.1.1), scikit-learn (version 1.5.2), and statsmodels (version 0.14.4) packages.

### Ethics, data sharing plan, funding, use of AI, and disclosures

This study was approved by our institutional review board (IRB approval: 2018P000688). The authors report no funding disclosures for this study. The investigation was performed at Massachusetts General Hospital, Boston, USA in accordance with the principles of the Declaration of Helsinki [22]. No generative AI tools were used for drafting and editing of the manuscript. Each author certifies that he or she has no commercial associations (e.g., consultancies, stock ownership, equity interest, patent/licensing arrangements, etc.) that might pose a conflict of interest in relation to the submitted article. Complete disclosure of interest forms according to ICMJE are available on the article page, doi: 10.2340/17453674.2025.43479

## Results

### Before matching

In the non-matched cohort of 265 patients, 55% (n = 147) were female, the mean age was 67 years, and the mean BMI was 26. In terms of primary tumor, 34% (n = 89) patients had slow-growth primary tumors, 31% (n = 81) had moderate-growth primary tumors, and 36% (n = 95) had rapid-growth primary tumors. The median total cost of prophylactically treated patients (1,229 CUs, IQR 1,189–1,320) was lower compared with patients treated for a completed pathologic fracture (1,548 CUs, IQR 1,459–1,635; MD –318, CI –599 to –31) (Table 1). Multivariate logistic regression analysis found no association between completed pathologic fractures and increased healthcare costs (OR 1.0, CI 0.8–1.2). However, an ECOG score of 3–4 was significantly associated with higher healthcare costs (OR 3.4, CI 1.7–7.0). Regarding surgical factors, endoprosthetic reconstruction (OR 1.3, CI 1.1–1.5) and the use of multiple implants (OR 1.9, CI 1.1–3.3) were linked to increased costs. Among laboratory values, lower hemoglobin levels (OR 0.8, CI 0.8–0.9) and higher white blood cell counts (OR 1.6, CI 1.1–2.5) were associated with increased costs. No other significant associations were identified for demographic, tumor histology, clinical presentation, or laboratory variables (Table 2, see Appendix).

**Table 1:** Costs of hospital stay and 30 days post-discharge for patients before (n = 265) and after matching (n = 200). Values are median (interquartile range [IQR])

Cohort Costs	Median (IQR) costs, CU		Median difference (CI)	P value
	Impending	Completed		
Non-PSM-matched cohort (n = 265)				
Patients, n	161	104		
Hospital stay	643 (581–687)	643 (581–685)	–172 (–264 to –46)	< 0.01
30 days post-discharge	100 (72–104)	52 (38–84)	32 (–73 to 86)	0.8
Total	1,229 (1,189–1,320)	1,548 (1,459 – 1,635)	–318 (–599 to –31)	0.02
PSM-matched cohort (n = 200)				
Patients, n	100	100		
Hospital stay	471 (460–487)	618 (599–697)	–25 (–152 to 159)	0.9
30 days post-discharge	84 (62–101)	69 (44–92)	31 (–74 to 88)	0.6
Total	1,551 (1,495–1,590)	1,508 (1,448–1,586)	44 (–294 to 262)	0.9

Costs are presented as cost-units (CU) which are the actual costs divided by a common denominator. Mann–Whitney U test was used for comparison of costs.

**Table 2:** Multivariate logistic regression results of increased healthcare costs

Variable	OR (CI)	P value
Demographics		
Male sex	1.06 (0.88–1.28)	1
Age	0.95 (0.87–1.04)	0.3
Body mass index	1.05 (0.96–1.15)	0.3
ECOG of 3 or 4	3.40 (1.65–6.99)	< 0.01
Additional comorbidity	0.89 (0.45–1.79)	0.8
Tumor histology		
Slow growth	Reference	
Moderate growth	0.86 (0.70–1.06)	0.2
Rapid growth	0.97 (0.79–1.19)	0.8
Type of surgery		
Intramedullary nail	Reference	
Endoprosthetic reconstruction	1.26 (1.05–1.52)	0.01
Plate-screw fixation	1.46 (0.92–2.29)	0.1
Dynamic hip screw	0.54 (0.14–2.09)	0.4
Multiple implants	1.89 (1.09–3.27)	0.02
Clinical presentation		
Pathologic fracture	0.98 (0.82–1.18)	0.8
Visceral metastases	0.95 (0.81–1.13)	0.5
Brain metastases	1.07 (0.84–1.37)	0.06
Previous systemic therapy	0.67 (0.33–1.35)	0.3
Laboratory values		
Hemoglobin	0.83 (0.75–0.91)	< 0.01
Platelet	0.93 (0.81–1.07)	0.3
Absolute lymphocytes	0.91 (0.79–1.04)	0.2
Absolute neutrophils	0.73 (0.47–1.12)	0.2
Creatinine	0.96 (0.88–1.05)	0.4
White blood cell count	1.63 (1.06–2.52)	0.03
Sodium	1.03 (0.94–1.12)	0.5
Calcium	1.05 (0.96–1.14)	0.3

High costs were defined as total healthcare costs over the course of the study period on the 75th percentile or higher. Regression was performed using purposeful regression methodology. All continuous variables were standardized to aid in interpretability of the regression analysis.

ECOG = Eastern Cooperative Oncology Group.

### After matching: primary outcomes

**Figure 3:**
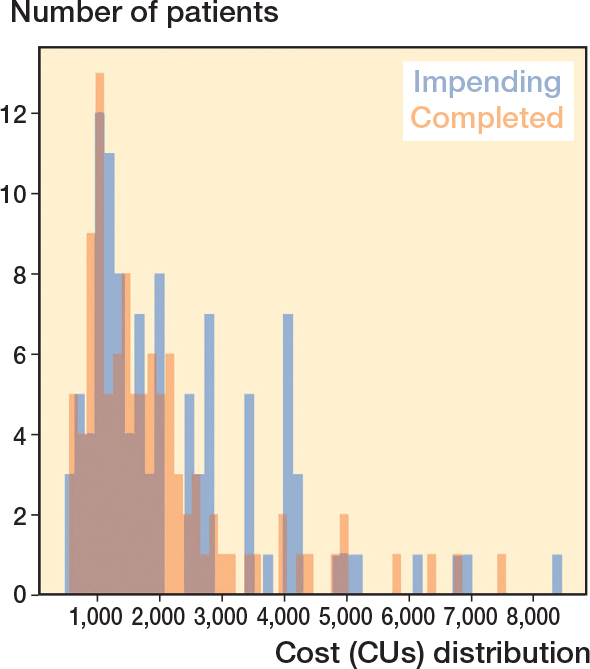
Cost distribution indicates a right-skewed pattern in total cost data.

After matching, all baseline variables were comparable between both cohorts (Table 3). For both groups, total treatment costs were right-skewed (Figure 3, see Appendix). The median total costs were not different between impending pathologic fractures (1,551 CUs, IQR 1,495–1,320) and completed pathological fractures (1,508 CUs, IQR 1,448–1,586; MSD –25, CI –152 to 159). Similar non-different patterns were seen when dividing the total cost into median cost during hospital admission for impending vs complete fractures (643 CUs, IQR 581–687 vs. 618 CUs, IQR 599–697; MSD –25, CI –152 to 159) and 30 days following discharge (100 CUs, IQR 72–104 vs. 69 CUs, IQR 44–92; MSD 31 CUs, CI –74 to 88) (Table 1). The confidence interval for the difference in treatment costs between the 2 groups excluded values greater than a 10% difference in cost units, suggesting that any observed cost difference is not clinically meaningful (Table 1).

**Table 3:** Patient baseline characteristics of the propensity score matched cohort (n = 200) in comparison with the non-matched (n = 265) cohort. Values are count (%) or median (interquartile range) unless otherwise specified

Variables	Non-matched cohort (n = 265)		SMD	Propensity score matched cohort (n = 200)		SMD
	Impending (n = 161)	Completed (n = 104)		Impending (n = 100)	Completed (n = 100)	
Age (SD)	67 (14)	68 (16)	0.05	68 (14)	68 (16)	
Female sex	95 (59)	52 (50)	0.18	45 (45)	48 (48)	0.14
Body mass index (SD)	26 (7)	26 (7)	0.57	26 (7)	26 (7)	0.08
Charlson comorbidity	71 (44)	59 (40)	0.43	48 (48)	38 (38)	0.28
Primary tumor growth			0.05			0.15
Slow	54 (34)	35 (33)		44 (44)	33 (33)	
Moderate	47 (29)	34 (33)		32 (32)	34 (34)	
Rapid	60 (37)	35 (33)		24 (24)	33 (33)	
ECOG score			0.22			0.09
0–2	131 (81)	75 (72)		77 (77)	73 (73)	
3–4	30 (19)	29 (28)		23 (23)	27 (27)	
Other bone metastases	120 (75)	76 (73)	0.99	72 (72)	73 (73)	0.02
Visceral metastases	77 (48)	65 (44)	0.09	40 (40)	45 (45)	0.10
Brain metastases	21 (13)	25 (17)	0.04	10 (10)	15 (15)	0.15
Previous systemic therapy	114 (71)	97 (66)	0.20	61 (61)	60 (60)	0.02
Type of surgery			< 0.01			0.07
Intramedullary nail	115 (72)	81 (55)	0.002	47 (47)	44 (44)	
Endoprosthetic reconstruction	37 (23)	55 (37)	0.003	50 (50)	49 (49)	
Plate and screw fixation	5 (3)	12 (8)	0.68	2 (2)	4 (4)	
Combination	4 (2)			1 (1)	3 (3)	
Laboratory values						
Absolute lymphocyte count (10^3^/µL)	0.9 (0.8–1.4)	0.9 (0.6–1.2)	0.05	0.9 (0.6–1.3)	0.9 (0.6–1.2)	0.13
Absolute neutrophil count (10^3^/µL)	5.5 (4.4–6.0)	5.5 (4.0–7.2)	0.55	5.5 (4.6–5.8)	5.5 (4.4–7.2)	0.25
Calcium (mg/dL)	9.3 (9.1–9.7)	9.3 (8.9–9.7)	0.09	9.3 (9.0–9.4)	9.3 (8.9–9.7)	0.09
Creatinine (mg/dL)	0.8 (0.7–1.0)	0.8 (0.7–1.0)	0.45	0.8 (0.7–0.9)	0.8 (0.6–0.9)	0.27
Hemoglobin level (g/dL)	11 (11–13)	11 (10–12)	< 0.001	12 (10–12)	11 (10–13)	0.19
Neutrophil-to-lymphocyte ratio	6.1 (3.7–6.8)	6.1 (4.9–9.4)	0.02	6.1 (5.2–6.9)	6.1 (4.8–7.8)	0.30
Platelet count (10^3^/µL)	250 (194–313)	250 (185–304)	0.60	250 (188–311)	257 (220–310)	0.07
Platelet-to-lymphocyte ratio	271 (187–322)	271 (220–413)	0.06	271 (206–395)	271 (227–411)	0.21
Sodium (mg/dL)	138 (137–140)	138 (136–140)	0.17	138 (136–139)	138 (137–140)	0.28
White blood cell count (10^3^/µL)	7.5 (5.8–8.7)	7.5 (4.8–10.3)	0.82	7.5 (5.4–8.4)	7.5 (5.1–10.1)	0.23

SMD = standardized mean difference; IQR = interquartile range; ECOG = Eastern Cooperative Oncology Group, 0–2: patients with better performance status, 3–4: patients with poorer performance status.

### After matching: secondary outcomes

After matching, no significant differences were observed in postoperative complications between the groups (Table 4).

**Table 4:** Overview of secondary outcomes length of stay, reoperations, and adverse events for patients before (n = 265) and after matching (n = 200). Values are count (%) or median (interquartile range)

Variable	Non-matched cohort		P value	Propensity score matched cohort		P value
	Impending (n = 161)	Completed (n = 104)		Impending (n = 100)	Completed (n = 100)	
Postoperative length of stay	4 (3–5)	4 (2–6)	0.7	4 (3–5)	4 (3–7)	0.2
Total length of stay	5 (3–8)	6 (4–9)	0.07	5 (4–9)	7 (5–12)	0.3
Discharge location						
Home	109 (68)	44 (42)	< 0.01	66	43	< 0.01
Rehabilitation facility	48 (30)	60 (58)	< 0.01	30	57	< 0.01
Other	4 (2.5)	0 (0)	0.5	4	0	0.1
Postoperative complications						
None	124 (77)	71 (68)	0.2	78	71	1
Wound infection	1 (0.6)	3 (2.9)	1	2	3	1
Wound dehiscence	0	2 (1.9)	1	0	1	1
Myocardial infarction	0	1 (1.0)	1	0	1	1
Sepsis	3 (1.9)	1 (1.0)	0.9	2	1	1
Delirium	2 (1.2)	7 (6.7)	0.01	2	7	0.3
Venous thromboembolism	14 (8.7)	7 (6.7)	0.3	8	2	0.1
Pneumonia	8 (5.0)	5 (4.8)	0.8	1	2	1
Other serious complications ^a^	17 (10)	11 (10)	0.3	7	5	0.8
Material failure	1 (0.6)	1 (1.0)	1	0	1	1
Reoperation	8 (5.0)	9 (8.7)	0.8	3	8	0.2
90-day mortality^b^	27 (17)	20 (19)	0.6	20	20	0.8
1-year mortality^c^	75 (46)	50 (48)	0.9	43	47	0.7

Costs are presented as cost-units (CU), which are the actual costs divided by a common denominator.

In the non-matched cohort, the Mann–Whitney U test was used for comparison of medians and the independent t-test was used for comparison of means. In the matched cohort, the McNemar test was used for categorical data and the Wilcoxon signed-rank test for continuous data.

a Including shock.

b 90-day loss to follow-up in non-matched cohort: 7/265, and in matched cohort: 4/200.

c 365-day loss to follow-up in non-matched cohort: 22/265, and in matched cohort: 15/200.

Moreover, we showed no differences in postoperative hospitalization duration (4 days, IQR 3–5 for impending fractures vs. 4 days, IQR 3–7 for completed fractures, P = 0.2) or total length of stay (5 days, IQR 4–9 vs. 7 days, IQR 5–12, P = 0.3) (Table 4). Patients treated for impending fractures were more often discharged directly to their home (66/100) compared with those treated for completed fractures (43/100, P < 0.01) and less often discharged to rehabilitation facilities (30/100 vs. 57/100, P < 0.01).

## Discussion

This is the largest healthcare expenditure data set in this patient population and PSM on 9 different variables. We aimed to compare prophylactic surgical treatment with treatment of completed pathological fractures in terms of total cost of care regarding patients’ femoral metastases. Cost analysis revealed that, after matching, patients who received prophylactic treatment incurred similar median healthcare related costs as compared with patients who underwent surgical treatment for a completed pathological fracture.

In terms of clinical outcomes, no significant differences were observed in the length of hospital stay, reoperation rates, or development of postoperative complications between the 2 groups. However, prophylactically treated patients were more likely to be discharged to their home (and less likely to require rehabilitation facilities).

Blank et al. reviewed 40 patients in 2016 and found that there was an economic benefit in treating impending pathological fractures compared with completed pathological fractures in long-bone metastases [23]. A similar study by Mosher et al. in 2019 found in 43,920 patients that prophylactic fixation was associated with lower total hospital charges and hospitalization length, leading to average savings of $3,405 per patient [24], while both studies suggested that there was an economic value in prophylactic stabilization.

A note of caution is due here, as these previous findings were based on a smaller sample size, and patients with metastasis comprised only a little over half of the study population [23]. In the study by Mosher et al., there was no adjusting for confounding factors. In our study, we initially enrolled 265 patients, almost 5 times as many as in previous literature utilizing institutional data. Furthermore, we adjusted for 13 confounders through PSM, adding to the study’s validity by creating a balanced cohort of 200 patients.

Our results are somewhat counterintuitive. Higher cost of care was expected in patients who underwent surgical treatment for a completed pathological fracture both during the hospital stay and following discharge. During the hospital stay, imaging and consultation required for an initial full workup of the disease and a generally longer length of stay would have contributed to higher costs in this cohort [3,5,23,24]. Additionally, patients admitted with a completed pathological fracture are not as prepared for the postoperative recovery and complication risks, nor had a chance for preoperative optimization, which we expected would summate to a higher cost of care postoperatively. This was reflected in the difference in discharge disposition between the 2 cohorts, with a higher proportion of patients with completed pathological fractures being admitted to a rehabilitation facility (see Table 4) that provides continuous monitoring but at a higher cost of care.

These results can be explained in part by the proximity of the postoperative outcomes between the 2 cohorts after matching for confounders. There was no difference in the length of stay between the 2 matched patient populations. There was also no difference in the rate of reoperation, complication, and survival, except for UTIs, which were more prevalent in the completed fracture group (see Table 3). This could imply that while the median cost of care of all 3 categories was lower in this study’s prophylactic treatment group, this difference was offset by the relatively similar postoperative length of stay and risk of complications between the 2 patient groups. Further regression analysis was performed to review the effect of factors initially included as confounding variables (Table 2, see Appendix). Surprisingly, laboratory values were associated with differences in cost of care, with hemoglobin (OR 0.83, CI 0.75–0.91) being inversely associated with higher costs of care, and white blood cell count (OR 1.6, CI 1.1–2.5) being associated with a higher cost of care. A higher ECOG (OR 3.4, CI 1.7–7.0) and use of multiple implants (OR 1.9, CI 1.1–3.3) were also associated with a higher cost of care (Table 2, see Appendix). The relationship between different laboratory values and healthcare costs could reflect the need for further intervention before or after undergoing surgery based on the health status of the patient. For example, a patient with a higher hemoglobin would be less likely to require perioperative transfusion. In contrast, a high white blood cell count could indicate the need for further workup and intervention to avoid postoperative adverse events [25-28]. However, it could be argued that both extremes of laboratory values may indicate further intervention and treatment planning that would contribute to the cost burden of surgical care. Further studies that evaluate these laboratory values in the context of this patient population are therefore recommended. These findings suggest that focusing on implementing solutions that reduce overall treatment cost for patients undergoing surgical treatment for an impending and completed pathological fracture would prove beneficial.

### Limitations

First, as a retrospective analysis, our data collection relied on archival information, which may have introduced selection bias. We had to exclude patients with missing cost data, which could impact the generalizability of our findings and potentially skew the observed treatment cost differences. Although our sample size was relatively large compared with other studies in this field, the absence of complete cost data might have limited our ability to detect a statistically significant difference in treatment costs between the two groups. However, baseline characteristics between the included and excluded patients were largely comparable, with standardized mean differences being small for most variables (Table 5, see Appendix). This suggests that the excluded patients did not differ meaningfully from the base cohort, minimizing the risk of selection bias due to missing data. Second, similar to many studies on this research area [3,5], we were not able to gather data on postoperative complications, reoperations, and associated costs that were addressed at outside hospitals and rehabilitation centers, possibly contributing to an underestimation of additional costs following discharge. For example, patients with completed pathologic fractures were more often discharged to their own homes instead of a rehabilitation facility or hospice. This could have impacted our results since these outcomes drive up the societal costs at large. Third, the choice of treatment was aided by the Mirels score, which is found to have considerable inter-observer variability [29]. Fourth, the methodological limitations of PSM warrant further discussion. Although PSM is increasingly utilized in healthcare cost-effectiveness research to address confounding, it requires rigorous implementation to ensure accurate propensity score estimation, careful matching, and thorough assessment of covariate balance. Despite these adjustments, PSM cannot replicate the randomization of a controlled trial and thus cannot fully establish causality. Additionally, PSM relies on observed covariates, meaning that any unmeasured confounders may still introduce bias into the results. As a result, while PSM helps mitigate some confounding factors, it remains an imperfect substitute for randomized controlled trials, which more directly test treatment algorithms and allow for stronger causal inferences. Fifth, this study is based on data from 2 tertiary referral institutions in the United States, which may increase the risk of selection and confounding bias and limit the generalizability of the findings. The healthcare practices, insurance systems, and patient management strategies in the United States may differ significantly from those in other countries, which could affect the applicability of these results internationally. For example, differences in healthcare access, the availability of prophylactic treatment, and follow-up care might influence outcomes and costs in other healthcare systems. Despite these limitations, the current study surpasses prior studies on this subject.

**Table 5:** Patient baseline characteristics of the base cohort (n = 265) in comparison with the patients who were excluded because of missing cost-data (n = 44). Values are count (%) or median (interquartile range)

Variables	Base cohort (n = 265)	Excluded due to missing data (n = 44)	SMD
Age	67 (59–74)	68 (59–75)	0.04
Female sex	147 (55)	26 (59)	0.10
Body mass index	26 (23–30)	24 (22–27)	0.17
Charlson comorbidity	111 (42)	13 (30)	0.31
Primary tumor type			0.07
Slow growth	89 (34)	13 (29)	
Moderate growth	81 (31)	13 (29)	
Rapid growth	95 (36)	18 (41)	
ECOG score			0.12
0–2	206 (78)	32 (73)	
3–4	59 (22)	12 (27)	
Other bone metastases	196 (74)	32 (72)	0.56
Visceral metastases	122 (46)	18 (41)	0.39
Brain metastases	36 (14)	5 (11)	0.07
Previous systemic therapy	178 (67)	24 (54)	0.26
Type of surgery			0.17
Intramedullary nail	159 (64)	20 (46)	
Endoprosthetic reconstruction	90 (34)	19 (43)	
Plate-screw fixation	9 (3)	3 (7)	
Dynamic hip screw	1 (0)	1 (2)	
Multiple operations	4 (1)	1 (2)	
Laboratory values			
Absolute lymphocyte count	0.9 (0.7–1.3)	0.9 (0.6–1.7)	0.19
Absolute neutrophil count	5.3 (3.8–7.3)	6.7 (4.5–9.5)	0.41
Calcium	9.3 (9.0–9.7)	9.5 (8.9–9.7)	0.12
Creatinine	0.8 (0.7–1.0)	0.7 (0.6–0.9)	0.41
Hemoglobin level	11 (10–12)	11 (10–12)	0.04
Neutrophil-to-lymphocyte ratio	6.1 (3.8–8.6)	6.1 (2.1–14.1)	0.35
Platelet count	252 (189–310)	261 (169–345)	0.08
Platelet-to-lymphocyte ratio	278 (182–366)	239 (153–403)	0.09
Sodium	139 (136–140)	138 (135–140)	0.13
White blood cell count	7.4 (5.5–9.3)	8.9 (6.7–13.0)	0.51

ECOG = Eastern Cooperative Oncology Group.

### Conclusion

We found no difference in hospital-related treatment costs between prophylactic surgery for impending fractures and surgery for completed pathological fractures after matching for confounding variables. However, our findings underscore the broader impact of completed pathological fractures on patient quality of life and healthcare resource utilization. Patients with completed pathological fractures were more likely to require discharge to rehabilitation facilities, suggesting that the societal costs associated with these fractures—including extended rehabilitation, reduced mobility, and loss of independence—are substantial. Prophylactic treatment offers a valuable opportunity to reduce patient morbidity and the downstream economic burden on the healthcare system.

*In perspective,* recent studies have demonstrated excellent performance of predictive models as a potential clinical tool in medicine, including orthopedic surgery [30-33]. Further studies could develop personalized models that predict patients who are at risk of increased rate of complication, adverse events, and higher care cost following surgical intervention for an impending or completed pathological fracture, which would supplement preoperative optimization and planning for postoperative care to reduce such outcomes.

## Notes

TMdG: conceptualization, methodology, formal Analysis, writing – original draft, writing – review & editing. MRS, DS: methodology, investigation, writing – review & editing. OQG: conceptualization, data curation, investigation, writing – review & editing. SJJ: formal analysis, writing – review & editing. MLF, ETN, KAR: resources, writing – review & editing. SAL-C: resources, data curation, writing – review & editing. JHS: conceptualization, supervision, writing – review & editing. PCJ: supervision, writing – review & editing.

Handling co-editors: Ilkka Helenius and Robin Christensen

*Acta* thanks Thomas Baad-Hansen, Aare Märtson and Michael Mørk Petersen for help with peer review of this manuscript.
